# Microblog Topic-Words Detection Model for Earthquake Emergency Responses Based on Information Classification Hierarchy

**DOI:** 10.3390/ijerph18158000

**Published:** 2021-07-28

**Authors:** Xiaohui Su, Shurui Ma, Xiaokang Qiu, Jiabin Shi, Xiaodong Zhang, Feixiang Chen

**Affiliations:** 1 School of Information Science and Technology, Beijing Forestry University, Beijing 100083, China; suxhui@bjfu.edu.cn (X.S.); msr16606386966@bjfu.edu.cn (S.M.); suxh85@bjfu.edu.cn (X.Q.); jiabin@bjfu.edu.cn (J.S.); 2 Engineering Research Center for Forestry-Oriented Intelligent Information Processing, National Forestry and Grassland Administration, Beijing 100083, China; 3 College of Land Science and Technology, China Agricultural University, Beijing 100083, China; zhangxd@cau.edu.cn

**Keywords:** earthquake emergency, effectiveness of topic-word, information classification, microblog, topic-words detection

## Abstract

Social media data are constantly updated, numerous, and characteristically prominent. To quickly extract the needed information from the data to address earthquake emergencies, a topic-words detection model of earthquake emergency microblog messages is studied. First, a case analysis method is used to analyze microblog information after earthquake events. An earthquake emergency information classification hierarchy is constructed based on public demand. Then, subject sets of different granularities of earthquake emergency information classification are generated through the classification hierarchy. A detection model of new topic-words is studied to improve and perfect the sets of topic-words. Furthermore, the validity, timeliness, and completeness of the topic-words detection model are verified using 2201 messages obtained after the 2014 Ludian earthquake. The results show that the information acquisition time of the model is short. The validity of the whole set is 96.96%, and the average and maximum validity of single words are 78% and 100%, respectively. In the Ludian and Jiuzhaigou earthquake cases, new topic-words added to different earthquakes only reach single digits in validity. Therefore, the experiments show that the proposed model can quickly obtain effective and pertinent information after an earthquake, and the complete performance of the earthquake emergency information classification hierarchy can meet the needs of other earthquake emergencies.

## 1. Introduction

Since 1980, China has been among the top five countries most frequently affected by damaging earthquakes [1]. After an earthquake, the affected areas are usually chaotic [2]. Therefore, an instantaneous efficient emergency management is required to develop plans and operations aiming to decrease casualties and losses [3]. Incorrect and inappropriate emergency responses can cause greater losses than the disaster itself [4]. The scientific nature and timeliness of the earthquake emergency decision-making depends on the acquisition and management of the disaster information, the emergency rescue information, the supply and demand information in the earthquake emergency support, and the social public opinion information [5,6].

Social media information can supplement the seismic monitoring data in earthquake emergencies [7]. In previous studies, researchers found that social information played a more important role than traditional methods in disaster awareness and determination [8,9,10]. Nearly real-time disaster information can be obtained from social media platforms, such as Twitter and Weibo (the largest Chinese microblog sites). Integrating social media information into earthquake decision-making can increase efficiency. The decision-making based on this information that comes from the public can provide public benefit and improve the public’s ability to prevent and mitigate disasters [11].

The earthquake-related information contained in microblogs is interactive, collected in real-time, and socially relevant. In recent years, data mining using data extracted from Weibo has been an interesting research topic, since short blogs contain minimal information and can cover both large and sparse areas [12,13,14]. Many studies on social media data collection, extraction, and analysis have been conducted to meet the requirements of natural disaster management, including earthquakes, floods, and typhoons [8,9,10,15,16,17]. On the Internet, earthquake disaster information is complex, randomly expressed, quickly disseminated, and spread by diverse carriers [18]. The automatic information acquisition from the Internet is the first step in the organization and management of earthquake emergency information. This process can be divided into manual extraction, semiautomatic extraction, and fully automatic extraction [19]. Web crawler is an automatic network information acquisition technology that can process as many network information pages as possible in a short time frame, but many problems exist, such as miscellaneous information, large system resource requirements, and excessive time consumption [20]. Crawler technology has been proposed to address these problems. A crucial part of a topic crawler consists in describing the topic content, which can improve the information processing efficiency, in order to describe the type of earthquake emergency from different angles according to the category. Relevant topics have been discovered by several methods, such as VSM (Vector Space Model) [21], ULW-DMM (an extended method combining Dirichlet multinomial mixture and user-LDA topic model) [22] and other methods. To address the earthquake characteristics, temporal extraction rules, location trigger dictionaries, and attribute trigger dictionaries have been created [23]. Analysing earthquake-related social media information usually requires separating buildings, green plants, transportation systems, water sources, and other classes to evaluate losses [16]. The classification of earthquake emergency information is mainly based on the object that is reflected by the information [24], the demand for information reporting [25,26], and computer storage and use [18,27]. The classification of seismic information is based on the usage of information. According to these studies, classification is the first step in applying data analysis [28]. However, few studies have been reported from the perspective of information collection. Crawling the earthquake-related information with few words can not accurately reflect the subject needs. In a crawler strategy, earthquake information needs to be searched based on earthquake-related keywords [29]. Therefore, we need to perform the classification not only from the perspective of analysis, but also from the perspective of information classification. However, an exhaustive literature review shows that the classification and information extraction from the perspective of public demand in the earthquake emergency response process have not yet been reported.

This paper focuses on topic detection after earthquakes and uses a cross-validation method to construct an information classification system for earthquake emergencies based on Sina microblog data. A subject word detection model is built to determine the different granularities of the word set of the earthquake emergency information classification subject. Microblog data following an earthquake are then taken as an example to verify the validity, timeliness, and application value of the model. Finally, we expect the model to improve the efficiency of earthquake emergency information processing and achieve efficient organization and management.

The remainder of the paper is organized as follows: Section 2 presents the data sources and data pre-processing. Section 3 details the classification hierarchy of the earthquake emergency information construction. Section 4 details the topic-words detection model based on the classification hierarchy. Section 5 applies the model and the hierarchy. Finally, conclusions are given in Section 6.

## 2. Data Sources and Data Preprocessing

### 2.1. Data Sources

The data in this paper comprise two parts: earthquake cases containing epicenters, occurrence times, magnitudes, and other additional parameters, which are labelled by the province where the epicenter is located and the serial number of the text; and microblog messages published after an earthquake by the public. The data were crawled with the keyword “earthquake” (in Chinese) on Sina Weibo. Sina Weibo has the same functionality as Twitter, expressing what the public sees and thinks in a timely manner [4]. With the development of the network technology and the popularity of mobile terminals, a large number of messages show instantaneously after an earthquake. In this study, raw messages are obtained using the keywords so as to quickly obtain the targeted information from a large amount of information. These obtained microblog messages include user ID, release time, microblog content (including pictures or videos), publishing location, microblog address, equipment source, etc. Information on the earthquake cases and microblog messages is shown in Table 1.

As mentioned earlier, the study tackles a topic-words detection model based on earthquake information classification hierarchy. Thus, the research is divided into three parts: the research and establishment of the hierarchy, the construction and verification of the detection model, and the application of the model. These three research contents have a chronological relationship. Therefore, in the sequel, all the information is divided into three data sets: data set I is historical data used in the first research contents, data set II is used in the second research contents, and data set III is newly gathered seismic data, aiming at practically applying the model. First, 01YG, 02SC, and 03XJ microblog information (hereinafter referred to as data set I) are used for the research and construction of the earthquake emergency information classification hierarchy. Second, 04YN microblog information (hereinafter referred to as data set II) is used to verify the efficiency of the earthquake emergency keyword detection model. Third, 05SC microblog information (hereinafter referred to as data set III) is used to apply the model and the classification hierarchy.

### 2.2. Data Preprocessing

**Figure 1:**
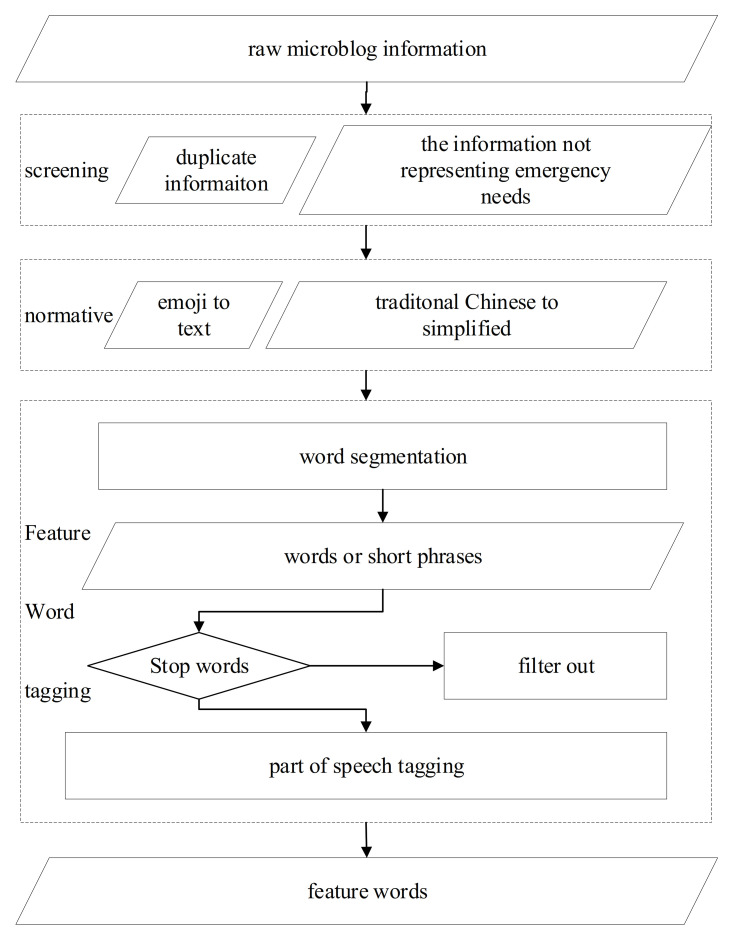
Data preprocessing flow chart.

Data preprocessing includes preliminary screening, text format normalization, and feature word tagging. In the preliminary screening phase, the information that belongs to earthquake news and topics that are initiated by the public are filtered out. In the format normalization phase, some elements that make the word segmentation difficult are treated. For instance, the traditional Chinese is converted to simplified Chinese, emojis are converted to text, etc. In the feature word tagging phase, messages are segmented using the ICTCLAS Chinese lexical word segmentation system (http://ictclas.nlpir.org/, accessed on 10 September 2012) Microblog messages were divided into words or short phrases and tagged with nouns, verbs, or phrases that can better express the subjects [30]. Stop words were filtered out, while the left words or short phrases that have been marked, are referred to as feature words. The data preprocessing flowchart is illustrated in Figure 1.

In Sina Weibo messages, the sentences and words that are put between the ‘#’ symbol define the topic, which is equivalent to the use of hashtags on Twitter [31]. In this study, messages containing the symbol ‘#’ were filtered out. The principles of preliminary screening and cleaning are shown in Table 2.

The microblog message related to the earthquake is interactive, real-time, social, and contains a lot of hidden value. Although the text is short, it holds a large amount of data with poor standardization. Due to the public’s habits and other reasons, some traditional Chinese characters often appear in microblogs. This causes some difficulties in the word segmentation process. In this study, ChineseConverter.dll in Visual Studio International Pack, is used to make this conversion. On the other hand, emoticons are often used to express feelings more conveniently. Therefore, emojis are converted in the preprocessing phase using the corresponding relationship between emoji and Unicode, as well as Unicode and characters. After word segmentation, the data will be traversed and stop words will be filtered out according to the stop words list. The nouns, verbs, quantifiers, numerals, and time words are then reserved according to the part of speech tagging.

## 3. Classification Hierarchy of Earthquake Emergency Information Construction

### 3.1. Process of Establishing the Classification Hierarchy

**Figure 2:**
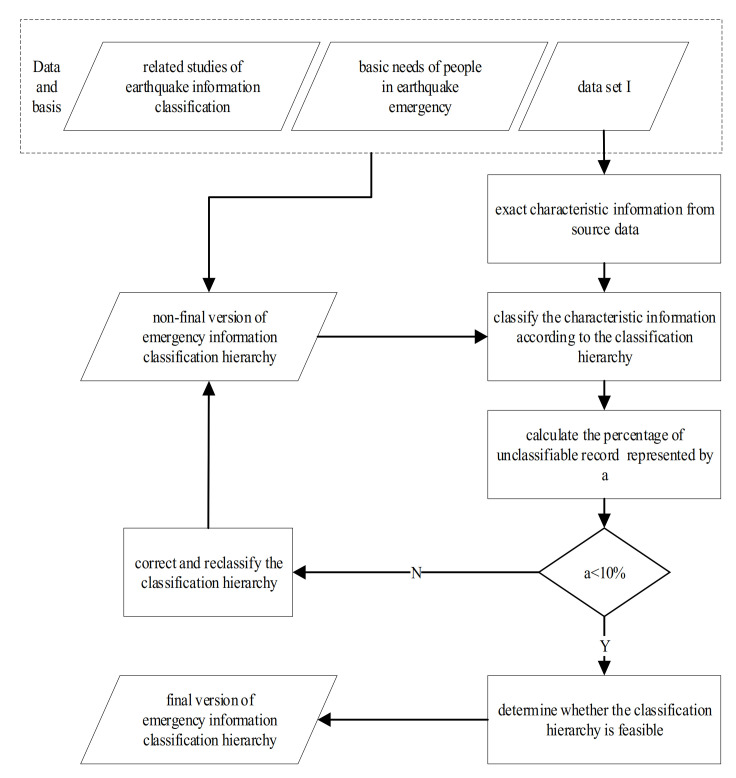
Construction process of the earthquake emergency information classification hierarchy.

According to the recent literature on earthquake information classification, this paper combines the basic needs of people in earthquake emergencies. People’s needs in earthquake emergencies consists in the basic life necessities, self-rescue, mutual-rescue, timely disaster information, psychological counseling, and other information about the earthquake. All this information need to be supported by the location information and time information. Therefore, the information is divided to location, time, disaster information, rescue, support, social opinion, etc. Considering the scientific aspects, applicability, and expansibility, the first edition of the earthquake emergency information classification hierarchy is constructed by feature words formed using data set I. The cross-validation method is then used to extract and classify the feature information. By repeatedly verifying the classification results, the threshold of the proportion for classification records is determined, while the classification hierarchy is corrected and reclassified according to the information classification results. K-fold cross validation is carried out by dividing the whole fitting data set into a training data set and a test data set. The fivefold cross-validation strategy can effectively reduce the computational cost in the modeling process, accelerate the sampling speed, and improve the modeling efficiency [32]. To ensure the scientific accuracy of the hierarchy, a fivefold cross-validation method is used. The establishment process of the earthquake emergency classification hierarchy is shown in Figure 2.

### 3.2. Earthquake Emergency Information Classification Hierarchy

The information classification highly affects the earthquake emergency system. Chinese researchers have made many efforts in this field. For instance, Guiwu Su classified the information into 17 categories [24], Dan Zhu classified the information into 9 categories in view of the application of a short message to report an earthquake disaster [33], and Man Dong classified the information from the command and decision-making of the emergency headquarters of the China Earthquake Administration [5]. In this study, by combining these classifications with the “Regulations on the Emergency Preplans for Destructive Earthquakes”, eight first-level indicators were obtained. Data set I was then divided with repeated verification and the percentage of the unclassifiable record was calculated. Having a percentage of unclassifiable records higher than 10% means that one tenth of the messages will not be classified. Therefore, the second-level indicators need to be corrected and the messages need to be repeatedly reclassified until the percentage becomes less than 10%, and the final version of emergency information classification hierarchy is constructed. According to the classification hierarchy establishment process, the classification hierarchy of the earthquake emergency information, including eight first-level indicators, 23 second-level indicators, and 29 third-level indicators, is finally constructed. The classification hierarchy has summarized the main categories of the earthquake emergency information, based on the microblog message. The second-level and third-level are completed from the perspective of establishing the scheduling relationship between the public’s demand and emergency services’ supply. All the indicators and the meanings of the lowest level indicators are given in Table 3.

## 4. Earthquake Emergency Information Hierarchy Topic-Words Detection Model

### 4.1. Topic-Words Detection Model Construction

**Figure 3:**
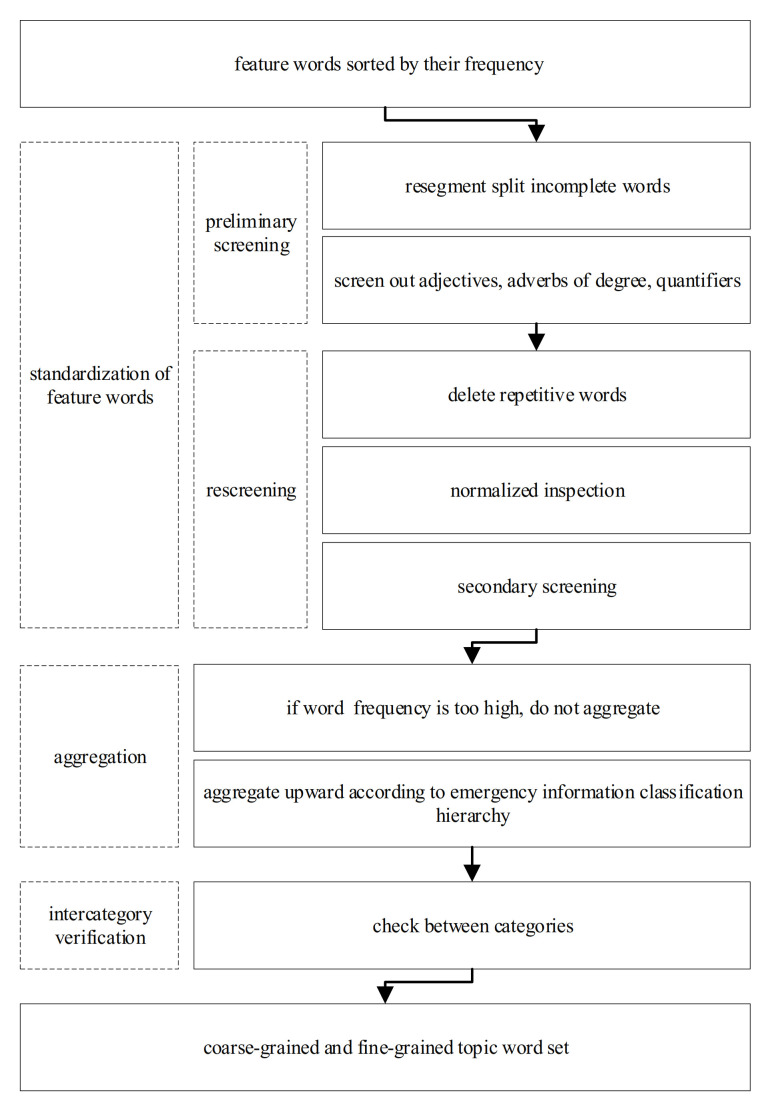
Data process used to detect the topic-words.

In the data preprocessing phase, feature words are obtained by word segementation and stop words filtering based on microblog messages. The word frequency statistics are first involved before building the model. Afterwards, the feature words are upward clustered according to Table 3 to obtain more concise topic-words. The coarse granularity and fine granularity topic-word sets are then obtained. Figure 3 shows the data process used to detect the topic-words, the model construction includes three key steps: the feature words standardization, aggregation, and inter-category verification.

#### 4.1.1. Aggregating Feature Words in the Same Category

Feature words aggregation is an upward aggregation process for words belonging to the same category under the earthquake emergency information classification hierarchy. Assuming that α and β are two feature words belonging to category C1, Q_α_ and Q_β_ are, respectively, the word frequencies of feature words α and β, α_i_ (i = 0 to n) and β_j_ (j = 0 to m), are the words or units constituting the feature words α and β. The word aggregation is performed as in Equation (1). If two different feature words contain the same unit and their meanings do not highly deviate, the repetitive one should be filtered out, while the other one will be kept for the next aggregation. K_α-β_ is one of the results that are achieved using Equation (1). Aggregation verification is then performed with the next feature word γ in the same category C1. Downward circulation is carried out until all the feature words in C1 are treated. Finally, the feature word set C1′ is the result obtained after aggregating C1.

#### 4.1.2. Checking between Categories

The third-level of the classification hierarchy of the earthquake emergency information is the lowest level. After the feature words under each third-level category are aggregated, it may appear that different categories contain the same feature words. Therefore, these feature words checked between the categories using Equation (2).

(1) $$Q_{\alpha }<20\cap Q_{\beta }<20\{\begin{array}{l} =1,\alpha_{x}=\beta_{y}\{\begin{array}{l} Y\;\mathrm{and}\;\alpha_{x}=\beta_{y}=K_{\alpha -\beta },\;\mathrm{let}\;\alpha =\beta =K_{\alpha -\beta }\\ N,\mathrm{aggregates}\;\mathrm{the}\;\mathrm{next}\;\mathrm{word}\;\gamma \;\mathrm{in}\;\mathrm{frequency}\;\mathrm{order} \end{array}\\ =0,\;\{\begin{array}{l} Q_{\alpha }\geq 20,\alpha \;\mathrm{no}\;\mathrm{polymerization}\;\mathrm{process}\\ Q_{\beta }\geq 20,\beta \;\mathrm{no}\;\mathrm{polymerization}\;\mathrm{process} \end{array}\;\;\;\;\;\;\;\;\;\;\;\;\;\;\;\;\;\;\;\;\;\;\;\;\;\;\;\;\;\;\;\;\;\;\;\;\; \end{array}\left(0\leq x\leq n,0\leq y\leq m\right)$$

(2) $$\theta_{x}^{C1^{\prime }}=\theta_{y}^{C2^{\prime }}\{\begin{array}{l} Y,\theta_{x}^{C1^{\prime }}=\theta_{y}^{C2^{\prime }}=\omega \{\begin{array}{l} \omega \;\mathrm{with}\;\mathrm{mark},\;\mathrm{recover}\;\mathrm{and}\;\mathrm{mark},\;\mathrm{then}\;\mathrm{return}\;\mathrm{to}\;\mathrm{the}\;\mathrm{set}\\ \omega \;\mathrm{without}\;\mathrm{mark},\;\mathrm{classify}\;\omega \;\mathrm{into}\;\mathrm{fuzzy}\;\mathrm{classification}\; \end{array}\\ N,\;\mathrm{end}\;\mathrm{the}\;\mathrm{checking}\; \end{array}\;\left(0\leq x\leq n,0\leq y\leq m\right)$$

In Equation (2), C1′ and C2′ are feature word sets of third-level classes in the earthquake emergency information classification hierarchy. $\theta_{i}^{C1^{\prime }}\left(i=0\;\mathrm{to}\;n\right)$ and $\theta_{j}^{C2^{\prime }}\left(j=0\;\mathrm{to}\;m\right)$ are the feature words of sets C1′ and C2′, respectively. After checking all the feature words between sets C1′ and C2′ based on Equation (2), the new sets C1˝ and C2˝ can be formed. The inter-category verification of other third-level classes is then continuously carried out until the interclass verification of all the classification feature word sets is completed.

#### 4.1.3. Constructing Coarse-Grained and Fine-Grained Feature Word Sets

Information collection and information management are the two main applications of subject words. Efficient information collection needs to ensure data comprehensiveness, which requires a large granularity of subject words. Information management requires accurate information classification, accurate data, and thus detailed and comprehensive granularity of subject words. Therefore, this study divides the feature words into different granularities according to their characteristics.

In Section 3, the feature words are divided according to the lowest level of earthquake emergency information classification hierarchy, which is a fine-grained set. Therefore, to meet the requirement of information collection, this paper focuses on a construction method for coarse-grained feature word sets. All kinds of fine-grained subject word sets, under the first-level category, are first merged together to form a new subject word set. The words with particularly low theme relevance are then eliminated, while aggregation processing is carried out on the same first-level category using the method shown in Equation (1). The verification steps are described in detail as follows:

First, *A*1 and *A*2 are two feature word sets under the first-level classification of earthquake emergency information. $\vartheta_{i}^{A1}\left(i=0\;to\;n\right)$ and $\vartheta_{j}^{A2}\left(j=0\;to\;m\right)$ are the feature words of sets *A*1 and *A*2, respectively.

If $\exists \;\vartheta_{i}^{A1}=\vartheta_{j}^{A2}=\sigma \;\left(0\leq i\leq n,0\leq j\leq m\right)$, then respectively filter out $\vartheta_{i}^{A1}$ and $\vartheta_{j}^{A2}$ from sets *A*1 and *A*2 in order to obtain sets *A*1′ and *A*2′. Feature words σ are denoted as $\sigma_{A1-A2}^{\prime \prime }$ and classified into the fuzzy feature word sets. All the words in sets *A*1 and *A*2 need to perform the same verification according to these operations.

If $\forall \;\vartheta_{i}^{A1}\neq \vartheta_{j}^{A2}\;\left(0\leq i\leq n,0\leq j\leq m\right)$, this ends the verification between sets *A*1 and *A*2. The next round of inter-category verification is continued until the verification of all the first-level classification feature word sets is completed.

#### 4.1.4. Fuzzy Feature Word Set Processing

The processing of the fuzzy classification feature word set follows the steps given in Table 4. The fuzzy classification feature word set is generated by inter-category checking (Equation (2)) and coarse–fine granularity feature word sets (the upper section). The sets need to be reclassified according to the definition of the classification indicator of the earthquake emergency information.

### 4.2. Model Validation

The validity *P* of the topic-word detection model is verified by comparing the timeliness and accuracy of information collection experiments without and with the classification topic-words. In the sequel, the information collection experiments without and with classified topic-words are referred to as ‘the former’ and ‘the latter’, respectively. The timeliness *T* and accuracy *R* are determined by two information collection experiments using Equations (3) and (4), whereas *T_b_* and *R_b_* are the time and accuracy for the former, and *T_a_* and *R_a_* are the time and accuracy for the latter. The effectiveness of the feature words is calculated using the number of all the information records *r* and the number of effective information records *q*, based on Equation (5). *P* is then calculated using Equation (6). When the timeliness and accuracy of the information collection are both higher than those of the former experiment, the proposed model is effective for actual application in earthquake emergencies.

(3) $$T=T_{b}-T_{a}$$

(4) $$R=R_{a}-R_{b}$$

(5) $$R_{i}=q_{i}/r_{i}\times 100\%\;(i=a,b)$$

(6) P=T×R

## 5. Case Analysis and Discussion

In the case analysis, information collection, information classification, and model verification tests are performed using the Python programming language.

### 5.1. Coarse-Grained and Fine-Grained Word Sets

Earthquake emergency information classification coarse-grained and fine-grained topic-word sets are formed by the proposed model, based on data set I. Part of the fine-grained and coarse-grained topic-word sets are shown in Table 5 and Table 6, respectively.

In the fine-grained topic-word sets, there are 140 words in the five levels of the first level: disaster investigation. In the coarse-grained topic-word sets, there are 93 words in the first level: disaster investigation. Various characteristics exist in the earthquake emergency, depending on the earthquake. Information collection according to fixed subject words may miss important information at the current stage, and this is not satisfactory to dynamic changes in the event [34]. Thus, the topic-word set should be updated in real time to meet the needs of the earthquake emergency information collection and classification. In addition, mining characteristic subject words for information collection helps to expand the dataset, to obtain more accurately captured earthquake information. This can support a reference for scientifically formulating earthquake emergency plans. Therefore, the topic-words validity will be tested from three aspects: the validity of information classification, the timeliness of information collection, and the completeness of the topic-word set.

### 5.2. Analysis of the Information Classification Validity

Information classification should be accurate and detailed. An information classification experiment is carried out using a fine-grained set and data set II to test the efficiency of the earthquake emergency information classification hierarchy. A record of microblog information often contains a variety of information categories. To collect comprehensive information for different categories, the study uses a multiple classification method, that is, if a record contains multiple categories of information, it will be divided into multiple simultaneous corresponding categories. Therefore, one message may be calculated more than one time in the validity records. The results of the experiment are shown in Table 7.

It can be seen in Table 7 that the proportion of disaster investigation—disaster situations, emergency support—traffic, location information, disaster investigation—abnormal phenomena and time information is more than 50%. This is the largest proportion. The second greatest proportion consists of the social public opinion, emergency rescue, and emergency support, while the disaster situation, location, time, and traffic information are the most concerned, followed by the emergency support, rescue, and social public opinion. By tracing back to the microblog data set, the earthquake location, time, disaster, and other circumstances are given the highest attention by the public, while the attention given to the emergency support and rescue is even higher. In addition, the news propaganda, and the social public opinion information, published by the major official media, also occupy a certain proportion. Using the set of classified topic-words as the standard for information classification is feasible. All the messages in data set II have been classified to these categories, in the classification hierarchy. This approach can implement the classification of earthquake emergency information and accurately perform information classification management.

### 5.3. Analysis of the Information Collection Timeliness

**Figure 4:**
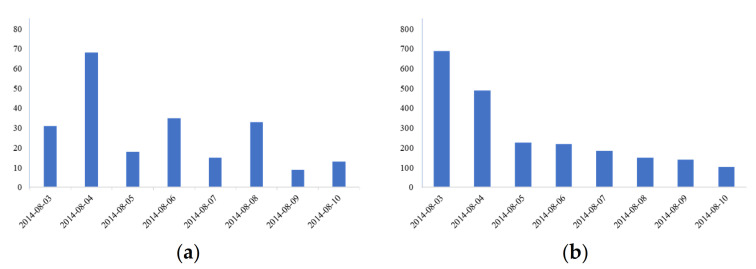
Daily amount of microblog messages; (**a**) used method *a*, (**b**) used method *b*.

The coarse-grained subject word set is selected for the information collection experiment, while considering the data comprehensiveness and accuracy. Taking the most sensitive emergency rescue information in the earthquake emergency work as an example, the general topic crawler method *b* and the topic crawler method *a,* based on classified topic-words, are used to capture the messages from earthquakes that occurred a week later. Other conditions of the two comparative experiments are consistent. The Ludian earthquake was taken as an example for information collection. In method *b*, microblog messages were crawled by the “earthquake” keyword, while the message time is between 2014-08-03 00:00:00 and 2014-08-11 00:00:00, which includes the time at which the Ludian earthquake took place, as well as the seven days that followed. In method *a*, microblog messages were crawled by the first-level A5 words that are used in the coarse-grained word sets as keywords. The message validity is judged based on the earthquake rescue information. The experiment results are shown in Table 8. The daily number of microblog messages obtained by the two methods are shown in Figure 4. Earthquake emergencies are time-urgent and of great importance. Therefore, timeliness and accuracy are required for earthquake emergency information collection.

According to model (6), *P* is greater than 0, the time of information collection in the latter is less than that of the former, and the information validity is higher. Therefore, we can conclude that the proposed model is effective. More precisely, the time and effectiveness of the experiment without using classified topic-words was 2470 s and 12.77%, respectively. After using classified topic-words, the collecting time *T_a_* and effectiveness *R_a_* are 1913 s and 96.96%, respectively. Note that *R_a_* is calculated by the number of effective information records *q* and the number of all information records *r.* Although the use of more keywords in method *a* is time consuming in the information collection phase, the keywords can focus on the collecting extents. In terms of timeliness, the latter experiment has a small advantage over the former experiment. The number of all the information records that are collected by method *a* is much smaller than those collected by method *b*. Using keywords can help to focus on the collected information. The latter experiment outperforms the former experiment in terms of accuracy. Finally, it can be concluded that the obtained results generally verify the effectiveness of the proposed model.

### 5.4. Analysis on the Validity of Information Collection Based on Topic-words

The previous experiment shows that the validity of a whole category is high. In this section, the validity of a single word is analyzed. The use of appropriate topic-words in collecting information is helpful to improve the effectiveness and accuracy of the subject word extraction [35]. The message and image analyses can provide effective support to the government and rescue organizations [36]. Therefore, a validity experiment is carried out using a single topic-word. For instance, the topic-word “evacuate” is used to crawl microblog messages. The crawler got 28 records, while 27 records represent the information about evacuation. Thus, the validity of the topic-word “evacuate” is 96%. The effective records of all 25 words are shown in Table 9.

It can be seen in Table 9 that the average validity of the 25 topic-words is 78%. The validity of several topic-words is over 80%, while some of them reach 100%. The information collection experiment shows that the overall cumulative validity of the set is 96.96%. Therefore, the emergency rescue topic-words of the coarse-grained set are effective.

However, some topic-words have low validity, such as “resume classes”, “victory”, “accident”, and “find” having a validity not exceeding 30%. The analysis shows that the total number of data records containing the words “resume classes”, “victory” and “accident” is less than 15. These words with less records also have less validity. In the Ludian earthquake, these topics do not reach such attention, and the messages that contain these words do not represent the same meanings. Therefore, these words can be removed from the word sets to meet the needs of the Ludian earthquake. However, for the comprehensive consideration of information acquisition, these words should not be removed from the word sets. In addition, the number of data records containing “find” is 100, but its validity is only 23%. This indicates that these words do not fit the corresponding earthquake, and thus we can determine whether to omit them or not. The experiment is carried out based on the 04YN, the Yunnan Ludian earthquake, and a coarse-trained rescue topic-word set in order to verify the effectiveness of the single words. The validity of other words can be tested using the same method. We can deduce, from the tested and evaluated results, that for each earthquake, the topic-word set should be slightly revised to meet the requirements of earthquake emergencies. In this case, more records with lower validity should be removed from the word sets to fit the Ludian earthquake. Moreover, topic-words having zero effective records, such as“resume classes”, should be removed. Finally, a small number of effective records with a high validity indicates that the public is giving less attention to the corresponding topics. The corresponding topic-words are then effective for collecting information, and they can represent the effectiveness of the word sets from another perspective.

### 5.5. Analysis of the Topic-Word Set Completeness

The completeness of the topic-word set is judged by the number of new words assigned with the earthquake. After performing the segmentation process using the ICTCLAS system, and the word frequency statistics based on data set II, the obtained top 20 words in word frequency order are shown in Table 10.

The word in the recommendation list will be further treated after topic-word detection. The new words that can accurately express the categories’ meanings will be added to the corresponding category.

A word with a specific seismic attribute will be added to a specific set to expand the data set. Words without actual meaning and that cannot accurately express the theme will be added to a secondary stop word set for filtering invalid data. An uncertain word will be added to the word set to be processed. It will be further processed considering other earthquakes. The top 20 words of the 04YN earthquake are processed and classified following this method. In the fine-grained word sets, the third-level earthquake situation has 13 words, the social mood-positive level has 57 words, and the non-emergency-support level has 7 words. After the treatment, 2 words will be separately added to the third-level. The results are shown in Table 11.

It can be seen from Table 11 that topic-word detection can extract new topic-words that express actual meanings in a short time. This approach can mine the seismic characteristic words as well as new unclassified words to detect subjects to focus on. Simultaneously, the words without actual meaning will be added to the stop word set to be further filtered out. According to the results, the number of newly added topic-words is 6. The new words can be added in a short time. Adding new words to the fine-grained word sets can improve and complete the word sets. As the data set continuously improves, topic-word collection can be effectively improved.

### 5.6. Hot Topic-Word Application

Topic-words can help the head of the emergency operations center in leading teams to efficiently coordinate emergency management responses [37] and reasonably allocate emergency resources [38]. In this study, we use the topic-word detection model to discover hot topic-words that would supply data support to the heads of the emergency operations center and teams.

**Figure 5:**
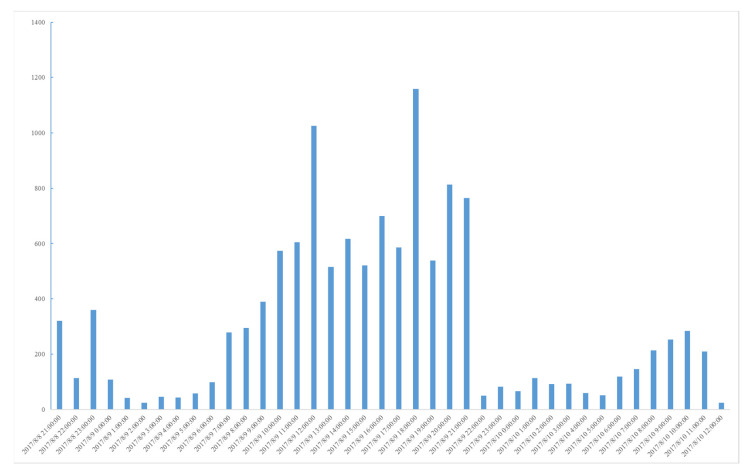
Message volume per hour of 05SC earthquake.

On 8 August 2017, Jiuzhaigou earthquake of magnitude 7.0, hit Sichuan Province in China. Its focal depth was 20 km. The earthquake case in this paper is called 05SC. This earthquake affected more than 0.17 million people, while most people in the disaster area, and out of it, wrote what they saw, heard, and felt on microblogs. Microblog message volume per hour is shown in Figure 5.

**Figure 6:**
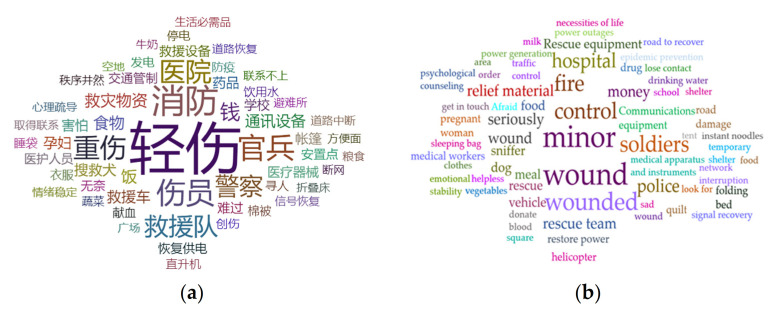
Word cloud of 05SC earthquake. (**a**) In Chinese. (**b**) In English.

We crawled the messages released from earthquakes that occurred 72 h after 05SC based on the Sina platform. The coarse-grained word sets are used to collect the earthquake emergency information. There were 16,166 related messages in total. By detecting the topic-words from the microblog information of 05SC, the topic-word cloud and the high-frequency words are shown in Figure 6.

In the word cloud, words with large font sizes and centered positions are the primary focus. The events that are not in the basic classification hierarchy can also be found in time. “Slight injury” (In Chinese “轻伤”) is the word having the highest frequency (1444). Some words that indicate rescue teams and organizations also appeared with a higher word frequency, such as “rescue teams”, “policeman”, “firefighter”, and “soldiers”. Simultaneously, more words with a positive social mood, such as “emotional stability”, and “in good order”, can be found. The word cloud shows the public’s sensitivity and observations after the earthquake. In the 05SC earthquake, the earthquake emergency measures and plans were improved due to the experience brought from previous major earthquakes.

Seventy-two hours after the Jiuzhaigou earthquake occurred, the words “food”, “tent”, “medical materials”, and “communication equipment” also had high frequency. These major rescue resources are usually deployed to the hardest hit areas following a major earthquake [39], which may lead to missing or delaying the implementation in some important areas. The topic-words frequency can help the earthquake emergency decision-making department in quickly focusing on the public needs and formulating a scientific emergency plan.

According to the frequency of the hot topic-words, the top 10 words are all in the word sets. Only “rescue car” and “sniffer dog” do not exist in the fine-grained word sets, in the top 20 words. These two words can be added to the word sets, and they can be classified to the emergency support level. Moreover, hot topic-words in the Jiuzhaigou earthquake demonstrate that the classification hierarchy shows a high completeness. Thus, there is no need to add new categories. Only two words need to be added to the existing word sets. Finally, the word sets generated according to the model can also be applicable to the earthquake.

## 6. Conclusions

Aiming to solve the problem of time-consuming information collection and a large amount of information processing, during an earthquake emergency response, this study classifies and organizes the information according to the actual needs of the earthquake emergency responses. The paper then constructs an earthquake emergency information classification hierarchy that includes 8 first-level and 29 third-level indicators. Based on the classification hierarchy of microblog data and timely microblog earthquake emergency information, the topic-word detection model is proposed. Afterwards, coarse-grained and fine-grained topic-words are built.

Taking the M6.5 Ludian earthquake of 3 August 2014 and the M7.0 Jiuzhaigou earthquake of 8 August 2017 as examples, only single-digit new words need to be added to the existing word set. The experiment shows that the classification hierarchy and the topic-word set constructed in this paper are relatively complete.

The rapid acquisition of earthquake information after an earthquake occurs is the key to earthquake emergency rescue [40]. The proposed method was compared with a method that only takes “earthquake” as a key word. The results show that the proposed method is faster than the former. It also leads to higher collected information effectiveness. This experiment verifies the effectiveness of the topic-word detection model. By applying the research model to the Lushan earthquake, the high-frequency topic-words after the earthquake can be obtained. This can provide data support for specific earthquake emergency rescue efforts. However, different words can have the same meaning. More precisely, the public can use different words to express the same senses on social platforms. For instance, the word “trapped” (in Chinese, “受困” and “被困”) represents a case where people cannot exit or escape, while words like “food” and “eat” do not have the same words or units, but the meaning is the same. Although in the aggregation phase, these words are considered by the same unit and semantic, this operation is manual. However, a thesaurus can be constructed to make these operations programmed. In a thesaurus construction, all the operations should be included to ensure the integrity of the topic-word set. Social media data can also be used to extract disaster-related tweets for earthquake emergency relief services [36]. However, in word frequency statistics, they need to be unified. The word semantics are key research topics in the application of subject words. In future work, we aim to study and analyze the word semantics. A synonym dictionary will then be constructed according to the characteristics of the earthquake management to highlight the words belonging to the same semantics, as much as possible in the statistics of the top words. This will be helpful in reducing the mistakes caused by neglecting different expressions. Deep research on the word semantics can also identify the most needed resources in the emergency phase to clearly and concretely guide emergency work.

## Figures and Tables

**Table 1:** Earthquake cases and microblog information table.

Earthquake Cases	Epicenter Location	Date of Occurrence	Magnitude	Focal Depth (KM)	Start Time ^a^	End Time ^a^	Messages ^b^
01YG	Yunnan-Guizhou border	2012-09-07	5.7	14	2012-09-07 11:19	2012-09-14 11:19	1515
02SC	Lushan, Sichuan	2013-04-20	7.0	13	2013-04-20 08:02	2013-05-20 08:02	83,456
03XJ	Hetian, Xinjiang	2014-02-12	7.3	12	2014-02-12 17:19	2014-02-19 17:19	176
04YN	Ludian, Yunnan	2014-08-03	6.5	12	2014-08-03 16:30	2014-08-10 16:30	2201
05SC	Jiuzhaigou, Sichuan	2017-08-08	7.0	20	2017-08-08 21:19	2017-08-11 21:19	16,166

^a^ The information is crawled, for each earthquake, by setting “earthquake” as the topic-word, the content of “start time” as the start time, and the content of “end time” as the end time; ^b^ The column “Messages” denotes the number of messages from the start time to the end time.

**Table 2:** Cleaning principles and processing methods for microblog information.

Objects	Operation	Reason
the messages contain ‘[]’	filter out	the symbol ‘[]’ is used to identify a news headline in microblog
the messages contain ‘#’	filter out	microblog’s title is usually placed between two ‘#’s to start a topic and attract users
characters such as line breaks and spaces	replace	no practical meaning and interfere with word segmentation
information such as ’Pupil Earthquake’	delete	similar but unrelated message
short text (less than 50 characters)	delete	no practical meaning for emergency work because of its length
duplicate information	filter out	different accounts reproduce exactly the same repeated information

**Table 3:** Earthquake emergency information classification hierarchy.

First-Level (A)	Second-Level (B)	Third-Level (C)	Meanings of the Third-Level
location information			longitude, latitude, and region
time information	publishing time		message publication time
	event time		time described in the message
disaster investigation	disaster situation	earthquake situation	description information such as magnitude and epicenter
		sense of earthquake	feelings during an earthquake
		casualties	casualty information
		destruction	damage caused by the earthquake
	abnormal phenomenon		abnormal phenomenon information accompanied by the earthquake
social public opinion	news propaganda	news notification class	news and notice information during earthquake
		news propaganda class	education and public information of earthquake emergency knowledge
	social mood	positive	information that contains positive opinion
		medium	pertinent emotional information published by people during earthquake emergency
		negative	negative remarks during the earthquake
	supervisory information		information on supervision, reporting and suggestions of relevant measures
emergency rescue	emergency		information about people in danger, including requests for and implementation of assistance
	disaster relief		relevant information on the treatment of secondary disasters and accompanying disasters
	emergency rescue situation		information about disaster relief progress and decision-making
emergency support	other material		material information that is difficult to classify
	warm	clothes	information about clothes
		cotton quilt	information of materials used against cold
	living	accommodations	information about materials related to accommodation, such as dispatching and accommodation locations
		shelters	information about public shelters, resettlement sites, etc.
	traffic	traffic information	road conditions, traffic control situations, dispatchable vehicle information, etc.
	food	ready to eat	information about food that is difficult to transport
		storable food	information about the food that is able to being stored and carried
	medical	medical staff	information about professional doctors and nurses
		medical equipment	information about medical and aid devices
		medicine	the status of medicines used for rescue
		blood bank	information related to rescue blood bank, blood donations, etc.
		injury situation	injury location, injury cause, etc.
		epidemic prevention	information related to disease protection
	community	electricity	information about electricity
		communication	information about the community
	safety and security		information about public security issues and security work
	rescue team	professional team	firefighters, etc.
		trained	information about NGOs, voluntary groups
		non-professional	unorganized and spontaneous rescue team information
	seek	searching	information for finding people and objects
		services	provides information about finding services
	psychological		information about psychological counselling and abnormal psychology
non-emergency	support		non-emergency donation or supply information
	other		other non-emergency information
comprehensive classes			fuzzy classification but of great significance to the information acquisition

**Table 4:** The proposed method for fuzzy classification feature word set processing.

Steps of the Method
1: *μ* is the marked feature word in fuzzy classification; E is one of the categories under the earthquake emergency classification hierarchy; *Q_μ_* is the word frequency of *μ*, and *δ* is the threshold for the coarse-grained and fine-grained sets;
2: if μ∈E, then classify *μ* into category *E*;
3: trace the word frequency of the words retained in the fuzzy classification, if $Q_{\mu }\leq \delta$, then classify *μ* to the comprehensive category of fine-gained feature word sets; otherwise classify *μ* to the comprehensive category of the coarse-gained feature word set;
4: finally, coarse-grained and fine-grained feature word sets are formed.

**Table 5:** Part of the fine-grained topic-word sets.

Coding for Category	Category Name	Topic-Words
A3B01C01	earthquake situation	magnitude, earthquake, strong earthquake, record, aftershock, disaster situation, disaster area, photo, earthquake situation, epicentre, disaster- stricken area
A3B01C02	sense of earthquake	tremble, degree, duration, sustained, fall, move, feel, wake up, shake, violent, shake, sharp, obvious, dazzle, crash, break, strong, intense, sound wave, sound, dizziness, fright, rattle, heartbeat, shock, silly, suffocation
A3B01C03	casualties	reports, deaths, destruction, population, numbers, killed, casualties, wounded, injured, lives, missing, died, damaged, dead, sacrifice, crush, earthquake death
A3B01C04	destruction	pulling down, scrapping, collapse, tragic situation, secondary disaster, subsidence, falling, breaking, ruins, landslide, destruction, in danger, boulder, collapse, lycopodium, crack, leakage of rain, falling objects, debris flow, broken, wall, gap, mountain, stone, damaged, rubble, tiles, dangerous houses, dangerous buildings, barrier lakes, smashing, serious disaster, earthquake damage, shatter, shock-off, destroy severely
A3B02C00	abnormal phenomena	wind, dog, flood, cooling, barking, thunder, visibility, temperature, precursor, frogs, lightning, moisture, cave-in, the sky, weather, temperature difference, fog, abnormal signs, fish, rain, cloud

**Table 6:** Part of the coarse-grained topic-word sets.

ID	Category Name	Topic-Words
A3	disaster investigation	magnitude, pulling down, scrapping, reports, collapse, tragic situation, tremble, degree, duration, sustained, fall, subsidence, move, tremble, breaking, ruins, wind, feel, dog, flood, landslide, wake up, shake, destruction, cooling, boulder, violent, barking, lycopodium, thunder, deaths, sharp, crack, obvious, dazzle, visibility, debris flow, crash, broken, temperature, precursor, strong, wall, frogs, gap, population, numbers, killed, mountain, lightning, lives, sound wave, sound, missing, moisture, stone, record, died, damaged, dead, the sky, weather, dizziness, rubble, tiles, dangerous, temperature difference, fog, sacrifice, subsidence, rattle, barrier lakes, abnormal signs, fish, cloud, smashing, photo, suffocation, destroy severely

**Table 7:** Results of the information classification validity experiment.

Categories	Records	Percentage
A1 location information	1870	85%
A2 time information	1267	58%
A3 disaster investigation—B1 disaster situation	2201	100%
A3 disaster investigation—B2 abnormal phenomenon	1347	61%
A4 social public opinion—B1 news propaganda	604	27%
A4 social public opinion—B2 social mood	791	36%
A4 social public opinion—B3 supervisory information	2	0%
A5 emergency rescue—B1 emergency	573	26%
A5 emergency rescue—B2 disaster relief	19	1%
A5 emergency rescue—B3 emergency rescue situation	866	39%
A6 emergency support—B1 other material	193	9%
A6—B2 warm	47	2%
A6—B3 living	756	34%
A6—B4 traffic	1924	87%
A6—B5 food	484	22%
A6—B6 medical	293	13%
A6—B7 community	444	20%
A6—B8 safety and security	4	0%
A6—B9 rescue team	540	25%
A6—B10 seek	21	1%
A6—B11 psychological	88	4%
A7 non-emergency—B1 support	127	6%
A7 non-emergency—B2 other	10	0%
A8 comprehensive classes	294	13%

**Table 8:** Results of the information collection timeliness experiment.

Method	Keywords	Time (s)	*r* ^a^	*q* ^b^
*b*	earthquake	2470	2201	281
*a*	trapped, deploy, evacuate, standby, in place, resume classes, recover, urgent, investigate, clear, rescue, repair, victory, accident, search, advance, orderly, find, support, execute, helicopter, command post	1913	230	223

^a^*r* represents the number of all the information records; ^b^ *q* represents the number of the effective information records.

**Table 9:** Results of the single topic-word validity experiment.

Topic-Word	All the Records	Effective Records	Validity
trapped	18	18	100%
deploy	7	6	86%
evacuate	28	27	96%
standby	18	18	100%
in place	11	3	27%
resume classes	5	0	0%
recover	25	10	40%
urgent	91	90	99%
investigate	4	4	100%
clear (in Chinese “排危”)	1	1	100%
clear (in Chinese “抢通”)	27	27	100%
rescue	46	46	100%
command post	50	50	100%
repair	21	21	100%
victory	15	4	27%
accident	10	2	20%
trapped	6	5	83%
evacuate	3	2	67%
search	5	4	80%
advance	6	5	83%
orderly	29	28	97%
find	110	25	23%
support	87	87	100%
execute	4	4	100%
helicopter	27	24	89%
total	654	511	78%

**Table 10:** Top 20 words in the recommended list of the Ludian earthquake.

Rank	Word	Part of Speech	Frequency
1	Ludian	NS	1298
2	tap	N	114
3	degree	Q	106
4	the Communist Youth League	NT	95
5	the dead	N	78
6	determination	V	74
7	formal	AD	73
8	depth	NS	70
9	huode	N	59
10	name	N	58
11	rest	V	58
12	pray	V	57
13	publish	V	52
14	fund	N	50
15	donation	V	49
16	sibling	N	46
17	zhaoyang	N	45
18	hand	N	45
19	help	V	44
20	century	N	43

**Table 11:** Decision of new topic-words in the Ludian earthquake.

Treatment	New Topic-Words and Their Determination
added to the fine-grained word sets	determination (classify to disaster investigation-earthquake situation), depth (classify to disaster investigation-earthquake situation), rest (classify to social public opinion-social mood-positive), pray (classify to social public opinion-social mood-positive), fund (classify to non-emergency-support) present
added to a specific set	Ludian
added to the secondary stop word set	degree, formal, name, publish, sibling, hand, help, century
to be processed	tap, the Communist Youth League, the dead, huode, zhaoyang

## Data Availability

All data, models, and code generated or used during the study appear in the submitted article.
